# Drought and freezing vulnerability of the isolated hybrid aspen *Populus x smithii* relative to its parental species, *P. tremuloides* and *P. grandidentata*

**DOI:** 10.1002/ece3.5364

**Published:** 2019-06-25

**Authors:** Nicholas J. Deacon, Jake J. Grossman, Jeannine Cavender‐Bares

**Affiliations:** ^1^ Department of Ecology, Evolution, and Behavior University of Minnesota St. Paul Minnesota; ^2^ Present address: Minneapolis Community and Technical College 1501 Hennepin Avenue Minneapolis 55403 MN; ^3^ Present address: Arnold Arboretum of Harvard University 125 Arborway Boston 02130 MA

**Keywords:** aspens, climate change, ecophysiology, electrolyte leakage, gas exchange, hydraulic conductivity, Niobrara National Scenic River, phenology

## Abstract

**Aim:**

We assessed the vulnerability of an isolated, relictual Pleistocene hybrid aspen population of conservation interest (*Populus x. smithii*) and the nearest populations of its parent species (*Populus grandidentata* and *Populus tremuloides*) to springtime post‐bud break freezing and growing season drought stress. Response to these stressors in the three taxa was compared in terms of avoidance and tolerance.

**Location:**

North American Midwest; USA.

**Methods:**

Unique genets from the hybrid Niobrara River population and from the two parental populations were propagated in a common garden from rhizome cuttings. We tracked their phenology before and after bud break and measured their vulnerability to freezing (stem electrolyte leakage and leaf chlorophyll fluorescence) and to drought (stem hydraulic conductance, leaf osmotic potential, stomatal pore index, and gas exchange).

**Results:**

*Populus grandidentata* was slower to leaf out, showed lower vulnerability to stem freezing and drought‐induced cavitation, but exhibited a lower capacity to tolerate drought stress through leaf resistance traits compared to *P. tremuloides*. Hybrids were similar to *P. grandidentata* in their overwintering strategy, exhibiting later bud break, and in their higher resistance to stem freezing damage, but they were more similar to *P. tremuloides* in their higher vulnerability to drought‐induced cavitation. The hybrids shared various leaf‐level gas exchange traits with both parents. All aspens showed limited loss of leaf photosynthetic function following moderate freezing.

**Main Conclusions:**

The Niobrara River hybrid population is vulnerable to drought due to its combination of inherited drought avoidance and tolerance traits. As climate changes, *P. x smithii* will likely suffer from increased drought stress, while being unaffected by frost during warmer springs. The two parental species contrast in their survival mechanisms in response to climatic stress, with *P. tremuloides* tending toward freezing tolerance but drought avoidance and *P. grandidentata* tending toward freezing avoidance and drought tolerance.

## 1 INTRODUCTION

Deciduous tree species face environmental challenges in continental environments, with freezing and drought representing two main stressors (Medeiros & Pockman, 2011; Menon, Barnes, & Olson, 2015). Species in temperate North America must be able to survive both freezing winter temperatures and seasonal drought stress. Adaptation to these abiotic forces has been critical in shaping the diversification of temperate tree lineages and the distribution of these species (Adams et al., 2009; Graham, 2011; Morin, Viner, & Chuine, 2008; Zanne et al., 2014).

Plants survive abiotic stress through some combination of tolerance and avoidance (Levitt, 1980; Savage & Cavender‐Bares, 2011). Tolerant plants can survive when their tissues are exposed to freezing temperatures or low water availability, and continue to photosynthesize through adaptations that prevent damage to cells and tissues. These include narrow vessels, antifreeze compounds, and morphological traits (e.g., torus–margo) (Baraloto, Morneau, Bonal, Blanc, & Ferry, 2007; Pittermann, Sperry, Hacke, Wheeler, & Sikkema, 2005). Avoidant plants also survive stress but do so by investing in mechanisms that shield them from stress or allow for temporary reduction in physiological activity, including closing stomata, shedding leaves, or delaying leaf out (Cavender‐Bares & Holbrook, 2001; Cavender‐Bares et al., 2005; J. Cavender‐Bares, 2007). Plants may have different strategies for different stressors; commitment to one strategy may limit a species’ access to another strategy and may also result in less growth or productivity (Koehler, Center, & Cavender‐Bares, 2012; Savage & Cavender‐Bares, 2013). Variation among populations in these strategies can thus result in variation in fitness in response to environmental stress, potentially leading to speciation (Heschel & Riginos, 2005).

**Figure 1:**
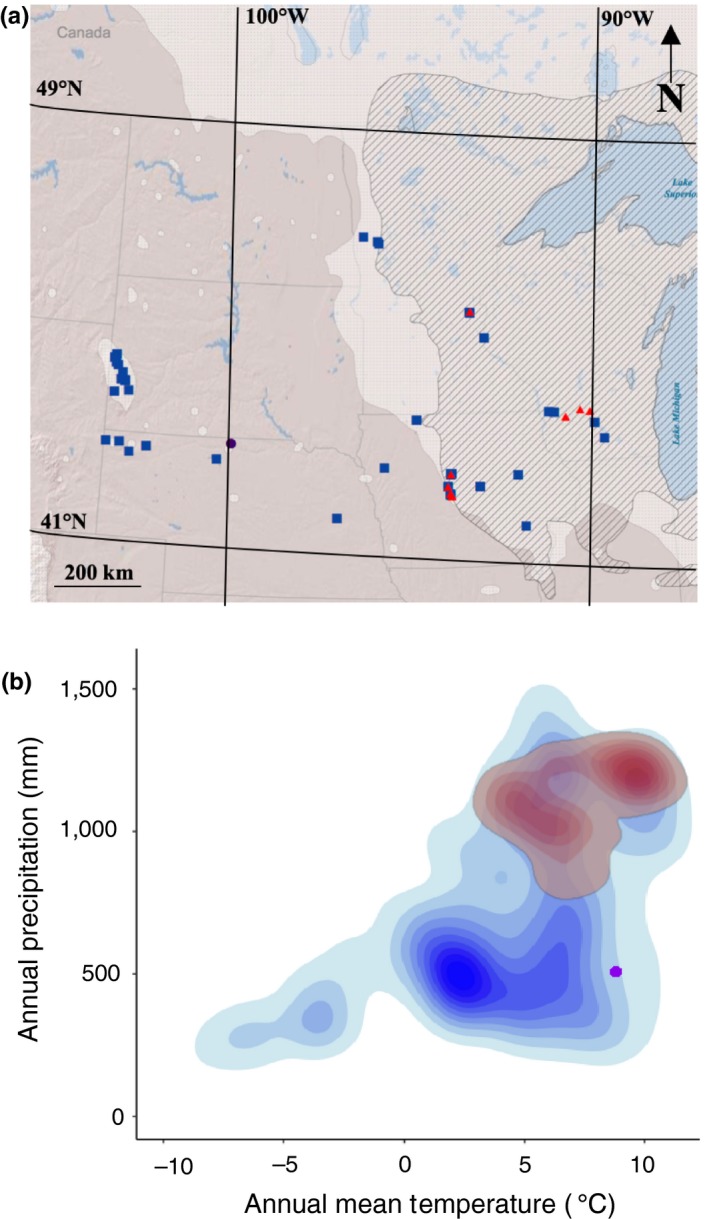
(a) Collections of *Populus grandidentata* (red triangles), *Populus tremuloides* (blue squares), and Niobrara *Populus x smithii* (purple circle) took place within and across the western boundary of the upper Midwestern ranges of *Populus grandidentata* (hatched) and *P. tremuloides* (light gray), and from isolated stands of *P. tremuloides*. Dark gray shading indicates areas where neither species is present. (b) Climatic (annual mean temperature and precipitation) envelopes of the ranges of *P. grandidentata* (red) and *P. tremuloides* (blue). A purple dot indicates the climatic conditions experienced by the hybrid *P. x smithii* population at Smith Falls. Details of climate envelopes in Appendix S3

The North American aspens are a widely distributed clade of temperate and boreal species that inhabit a range of environments across the continent, including eastern deciduous woodlands, Midwestern prairies, boreal parklands, alpine environments, and arctic environments. The sister species *Populus tremuloides* and *Populus grandidentata* are the only two North American representatives of the section *Populus* (*Leuce*) Duby. *Populus tremuloides* is the most widespread tree species in North America and inhabits ecologically diverse environments; *P. grandidentata* is confined to the mesic eastern United States and Canada. The two co‐occur across the range of *P. grandidentata* and have been known to hybridize (*Populus x. smithii*) occasionally in this region (Figure 1a). One hybrid population in particular, formed in northern Nebraska, USA during the last glacial maximum, is the focus of this study. It has persisted in isolated north‐facing canyons along the Niobrara River (Kantak, 1995; Kaul, Kantak, & Churchill, 1988) and lies well outside of *P. grandidentata*'s current range. Because it is part of a unique community of boreal species along a U.S. National Park System designated National Scenic River, land managers are committed to preserving this culturally and ecologically valuable stand of relictual aspens, comprising a dozen groves consisting of just three unique genotypes (Deacon, Grossman, Schweiger, Armour, & Cavender‐Bares, 2017). The population is thought to be in decline due to a variety of stressors (Robertson, Cahlander‐Mooers, & Dixon, 2018), potentially including the risk of post‐bud break freezing and drought concomitant with climate change.

In order to draw insights relevant to the Niobrara River *P. x smithii* stand's response to future climate change, we compared genotypes of the hybrid trees—in terms of their vulnerability to freezing and drought stress—to the nearest populations of their parental species from across the Upper Midwest. All three taxa were grown in a common garden in central Minnesota and in a controlled greenhouse environment. We observed the phenology before and after bud break and measured vulnerability to stem and leaf freezing of the hybrid and both parents in the common garden. This allowed us to compare their responses under the same environmental conditions to milder winters and experimentally simulated post‐bud break freezes, which may be expected to occur with increasing frequency as a consequence of climate change. We also assessed stem and leaf responses to drought stress, which is also predicted to occur in some climate change scenarios for the region (Mathys, Coops, & Waring, 2017).

Based on their distributions (Figure 1a), *P*. *tremuloides* experiences more variable environments (Figure 1b). If exposure to climatic variation results in greater plasticity (Miner, Sultan, Morgan, Padilla, & Relyea, 2005), we would expect *P. tremuloides* to exhibit greater plasticity and trait variability than *P. grandidentata*. Given the low temperatures *P. tremuloides* and *P.  grandidentata* are both exposed to within their ranges, we would also expect stems of both species to survive severe freezing, either through freezing tolerance mechanisms or by freezing avoidance mediated by the timing of spring bud break and leaf development phenology, which determines the exposure of leaves to freezing temperatures. The range of *P. tremuloides* also extends into drier environments than *P. grandidentata* so we would expect differences between them in the mechanisms for dealing with water stress in their stems and leaves and their response to simulated drought. Hybridization in plants offers natural experiments that have long interested evolutionary ecologists (Abbott, 1992; Anderson, 1948; Barton & Hewitt, 1985; Ellstrand, Whitkus, & Rieseberg, 1996; Rieseberg & Carney, 1998), and our research adds to the growing body of evolutionary ecological research in this genus. The genetic composition of *Populus* hybrids, for example, has been studied in the context of speciation, reproductive isolation, and adaptation (Christe et al., 2016; Gramlich & Hörandl, 2016; Jiang et al., 2016; Lexer, Fay, Joseph, Nica, & Heinze, 2005). In a previous study (Deacon et al., 2017), we found that the Niobrara hybrids shared nuclear genetic material from both parents and inherited the cytoplasmic genome from *P. grandidentata*. Given this background, we expected that the hybrids would share physiological freezing and drought vulnerability with both parents, and potentially be more similar to *P. grandidentata*.

## 2 METHODS

### 2.1 Sampling locations

From May 2013 to June 2015, we collected rhizome cuttings from aspen populations throughout the Midwestern U.S. range of *P. tremuloides* and *P. grandidentata* (Figure 1a; Appendix S1). Collections of *P. grandidentata* represent the species’ western range edge in Minnesota and Iowa and interior populations in Minnesota and Wisconsin. Cuttings of *P. tremuloides* were collected from these sites as well as from small, isolated stands in Nebraska and from the South Dakota Black Hills. Finally, we collected cuttings from the hybrid *P. x smithii* aspens in the Niobrara River Valley (NRV) of north‐central Nebraska (Deacon et al., 2017). Though this hybrid can and does form across its parents’ overlapping ranges (Barnes, 1961; Barnes & Pregitzer, 1985), we were, in the work reported here, interested in the physiology of the NRV hybrid aspens. We therefore confined our sampling of hybrid trees to this stand. Due to the few remaining stands of the NRV hybrids, and the attempt to collect from the nearest parental species populations in all directions surround the NRV hybrids, many more collections were made of *P. tremuloides*, which occurs in closer proximity to the NRV than of *P. grandidentata* and *P. x smithii*.

### 2.2 Common garden design and establishment

We established a common garden with all three aspen taxa grown out from collected rhizomes in the greenhouse. When collected, rhizomes were trimmed to 10–50 cm in length, transported to the University of Minnesota Plant Growth Facilities (St. Paul, MN), and propagated following root cutting protocols ((Luna, 2003); further description in Deacon et al., 2017). Our common garden was located in at Cedar Creek Ecosystem Science Reserve (CCESR, East Bethel, MN; 45°40’ N 93°10’ W), a Long‐Term Ecological Research Site. The garden was located within the interior of the current ranges of both *P. tremuloides* and *P. grandidentata* and both species could be found occurring naturally within 0.5 km. The garden site has excessively drained, sandy soils, and a humid continental climate (mean annual temperature: 6.2°C, mean annual precipitation: 782 mm). Minimum winter temperatures average −15°C, and, though periodic summer droughts do occur, our common garden was located in a mesic site adjacent to a slough and wetlands.

Trees produced in the greenhouse were randomly planted with respect to genotype at 1 m^2^ intervals in a cleared field that had been fenced to exclude deer. We watered all seedlings and mowed the space between them following establishment. Propagation success was variable among taxa and more of the *P. grandidentata* and *P. x smithii* stems failed than *P. tremuloides*. We transplanted 362 *P. tremuloides*, 42 *P. grandidentata*, and 31 *P. x smithii* stems for a total of 435 stems into the common garden. The number of physiologically and genetically distinct individuals of each species included in measurements and experiments below is given in Appendix S2.

### 2.3 Freezing experiments

#### 2.3.1 Leaf phenology

We recorded the status of overwintering leaf buds to monitor expansion, bud break, and leaf maturation weekly from late March to June 2016. Bud stage development was recorded for 13 *P. grandidentata*, 24 *P. x smithii*, and 292 *P. tremuloides* stems that had survived the transplanting and overwintering periods. Developmental stage was scored on a 0–5 scale from dormant to fully expanded leaves. In this scale, 1 indicated bud swelling, 3 indicated leaf emergence, and 5 indicated full expanded, green leaves.

#### 2.3.2 Greenhouse bud break

We collected branch segments containing at least 5 buds each from 10 naturally occurring mature *P. tremuloides* and 10 naturally occurring mature *P. grandidentata* trees near our common garden at CCESR on 14 January, 4 February, and 25 February 2016. These collections throughout the winter allowed for comparisons between the parental species on chilling length, and these dates correspond to approximately 91 days, 112 days, and 133 days from the first frost. Branches were placed in buckets of water that were periodically filled or replaced and brought into the University of Minnesota Plant Growth Facilities greenhouse under 15 hr of light per day and 20°C to force bud break. Buds on the branches were monitored every 3 days, and the date of bud break was recorded for each branch from each collection to compare the effect of winter length on bud break (Nanninga, Buyarski, Pretorius, & Montgomery, 2017).

#### 2.3.3 Stem freezing—electrolyte leakage method

Stem segments without attached leaves or branches were collected from common garden plants while dormant (21 March 2016), in early spring (17 May 2016), and in late fall (10 October 2016). Clonal replication was avoided by sampling from unique genotypes of each of the 3 species (Deacon et al., 2017).  A 10 cm segment from each of 12 *P. grandidentata*, 5 *P. x smithii*, and 38 *P. tremuloides* individuals was collected and placed in a floral tube filled with distilled water. Collected stems were kept cool and dark until processed in the Cavender‐Bares laboratory at the University of Minnesota. Four pieces, each 1 cm in length, were cut from each stem and placed in tubes filled with 9 ml nanopure H_2_O and 1 steel shot ball and assigned randomly to control, −5°C, −10°C, and −15°C freezing treatments. Control stem tubes were kept at 4°C for the duration of the freezing process. We used a programmable temperature controller with a circulating pump (Cole‐Parmer) in conjunction with a modified chest freezer to control the temperature and keep the plants in the dark. Temperatures were programmed to decline at 5°C per hour and hold for 30 min at −5°C, −10°C, and 15°C. Samples were removed for each temperature after the 30‐min hold. Pre‐ and postautoclaved conductivity of the control and freezing treatment groups were measured using a conductivity probe (Oakton 510; Oakton Instruments) following a modified protocol from Friedman, Roelle, Gaskin, Pepper, and Manhart (2008) and Koehler et al. (2012). An index of injury (%) was calculated to compare the electrolyte leakage caused by cell rupture at different freezing temperatures compared to the boiled electrolyte leakage (Flint, Boyce, & Beattie, 1967). Freezing tolerance was calculated as 100 less the index of injury such that positive values indicate higher tolerance.

#### 2.3.4 Leaf freezing—chlorophyll fluorescence method

Branches with at least 2 attached leaves were collected from common garden plants for measurements of leaf chlorophyll fluorescence postfreezing. Injury to leaf tissue as indicated by a decline in photosynthetic function due to freezing can be quantified by variable chlorophyll fluorescence (Boorse, Bosma, Meyer, Ewers, & Davis, 1998; J. Cavender‐Bares, Apostol, Moya, Briantais, & Bazzaz, 1999, p.; Feild & Brodribb, 2001; Cavender‐Bares et al., 2005, p.). Fully leafed‐out branches from the same 59 plants as used in the electrolyte leakage experiments were collected for both control and freezing treatments on June 7, 2016. All branches were collected by clipping stems under water and the cut ends were kept in floral tubes filled with distilled water to maintain leaf hydration. Collected branches and leaves were kept in darkness at 20°C for 24 hr prior to beginning treatments. The experimental group branches and leaves were placed in the freezer and cooled at the same rate as in the electrolyte leakage experiment to −7°C. The temperature was selected based on earlier trials that revealed when a freezing response could be detected as well as consideration of the possible late‐spring freezing temperatures plants might experience in the field. The consequences of freezing for photosynthetic function were assessed by measuring the maximum quantum efficiency (in the dark) using a pulse‐modulated fluorometer (Hansatech Instruments); percent decline in Fv/Fm was calculated relative to the control value measured at four time intervals over 24 hr.

### 2.4 Drought responses

#### 2.4.1 Stem hydraulic conductance, native embolism, and stem vulnerability to cavitation

Stem hydraulic conductance and native stem embolism were inferred from the flow rate of a 20 mM KCl solution of nanopure water through stem segments collected by predawn cutting of common garden plants underwater on 27 July 2016. 15‐cm‐long segments from 11 *P. grandidentata*, 6 *P. x smithii*, and 10 *P. tremuloides* were placed in floral tubes in the dark and processed in the Cavender‐Bares laboratory at the University of Minnesota. Predawn leaf water potential (Ψ_PD_) was measured on leaves of the same trees prior to stem collection using a pressure chamber (PMS Instruments). Leaves on the collected stems were removed, and leaf area of all leaves distal to the segment was quantified. Axillary leaf scars were covered with superglue (LocTite Gel), and stem diameter was recorded prior to placing stems in a tubing apparatus that allowed the solution to move gravimetrically under a known pressure gradient (Sperry, Donnelly, & Tyree, 1988). Flow rate was determined by an electronic balance connected to a computer. The hydraulic pressure head was maintained at approximately 5 kPA. Before each measurement, background flow was verified to be zero. Measurements were taken after approximately 3 min, after steady flow rate was reached. After the initial measurement was taken for each stem, branches were flushed with the solution for 2 min at approximately 150 kPa using a mechanically operated syringe pump.

Vulnerability to tension‐induced cavitation was compared among the three taxa by spinning the stem segments at increasing angular velocities in a centrifuge (Sorvall Model RC‐5B; Ivan Sorvall Inc) fitted with a modified rotor (Alder, Pockman, Sperry, & Nuismer, 1997; Pockman, Sperry, & O'leary, 1995). The centrifuge was calibrated for angular velocity to allow conversion to units of tension (Holbrook, Burns, & Field, 1995). Stems were removed from the tubing apparatus and placed into a water‐filled cap that created a meniscus around the ends of the stems while spinning. Tensions were created by spinning at faster speeds for 6 min each and measuring the flow of the solution through the stems after each spin. Percent loss of conductivity was calculated by comparing flow rates after each spin to the flushed flow rate.

#### 2.4.2 Gas exchange and water use efficiency

Gas exchange was measured on two to three leaves per unique genotype in the common garden plants, including 57 *P. tremuloides*, 14 *P. grandidentata*, and 4 *P. x smithii* individuals. Plants were measured at midday (1,200–1,400) on 12–14 September 2016 using a portable photosynthesis system (LICOR 6,400–40, Licor, Inc) at ambient CO_2_ (400 ppm), VPD, and light intensity of 1,500 mmol m^−2^ s^−1^. Intrinsic water use efficiency (WUEi) was calculated as light saturated photosynthetic rate (*A*_max_; µmol m^−2^ s^−1^) divided by leaf stomatal conductance (*g*_s_, mol m^−2^ s^−1^).

#### 2.4.3 Leaf osmotic potential

Leaf osmotic potential was determined from leaf disks from fully saturated plants following a modified protocol from (Bartlett et al., 2012). 6.35‐mm‐diameter leaf disks were cut using a paper hole punch from the same set of common garden plants as gas exchange and SPI measurements on the mornings of 1 August 2016 and 2 August 2016. Tubes containing the leaf disks were immediately placed in liquid nitrogen. We removed the leaf disks from the liquid nitrogen and placed them in humidified plastic bags to thaw for approximately 5 min before disrupting them with 10–12 pin holes and placing them one at a time into the VAPRO Vapor Pressure Osmometer Wescor, Vapro 5,520). Osmometer readings in mmol/kg (*x*) were converted to MPa based on the van't Hoff relationship at 20°C (Ψ _π_ = (−2.437 × 10^−3^ m^3^ MPa mol^−1^ K^−1^)K x). The osmometer was calibrated with standards obtained from Wescor Inc.

#### 2.4.4 Leaf stomatal pore index

Three to five leaves per plant were collected from the same plants as gas exchange measurements. Stomatal densities and guard cell lengths were determined from clear nail varnish impressions on the abaxial leaf surface between the mid‐vein and the leaf margin and measured on images taken from a microscope camera using ImageJ software (Rasband, 1997). Measurements were averaged over three regions of the impression at 20× and 40× magnification, respectively. Total stomatal pore area index (SPI; a dimensionless index of stomatal pore area per lamina area) was calculated as stomatal density × guard cell length^2^ (Sack, Cowan, Jaikumar, & Holbrook, 2003).

### 2.5 Data analysis

For each set of measurements or experiments described above, we used a two‐step linear regression approach to determine the factors affecting aspen physiological performance. First, we used bivariate models to assess whether there was an effect of taxonomic identity (*P. grandidentata*, *P. x smithii*, or *P. tremuloides*) on physiological performance. Then, because *P. grandidentata* and *P. x smithii* represent a restricted range of states and climates (mesic climates in only three and one states, respectively) relative to *P. tremuloides*, we constructed a second set of multiple regression models for *P. tremuloides* only. These models predicted physiological performance based on state of origin (IA, MN, NE, SD, or WI; these sometimes served as surrogates for latitude and longitude of collection) or climate effects (relevant climate variables downloaded from Climate WNA) (Wang, Hamann, Spittlehouse, & Carroll, 2016)). In particular, we considered annual heat moisture index (AHM; mean annual temperature divided by mean annual precipitation) and number of frost‐free days (NFFD) as potential climatic predictors of physiology. When repeated measures were taken on a plant over time or at different freezing temperatures, this information was also included in regression models, including interaction effects when they improved model fit. In all cases, we compared hierarchical models using likelihood ratio tests and nonhierarchical models using AIC tests and present findings from the best‐supported, most parsimonious model for each measurement or experiment. When significant ANOVAs indicated differences among levels of categorical predictors, we used post hoc tukey tests (“HSD.test” in agricolae; (De Mendiburu, 2016)) to differentiate between factor levels at α = 0.05.

Curves representing loss of stem hydraulic conductance with decreasing water potential were fit with a Weibull distribution using the *fitplc* package (Duursma & Choat, 2017). PLC_50_ values, which give the water potential at which 50% of hydraulic conductance is lost, were extracted from these curves and confidence intervals were generated using bootstrapping. We also use ANOVA to assess differences among taxa in predawn water potential, native stem embolism (before spinning), and the PLC reached by each individual at the minimum water potential imposed (−2.2 MPa). Because sample sizes for this set of measurements are low, we did not model the consequences of state or climate of origin for stem hydraulic conductance properties. All data were analyzed in R (R Core Team 2013, v. 3.4.1).

## 3 RESULTS

### 3.1 Phenology

**Figure 2:**
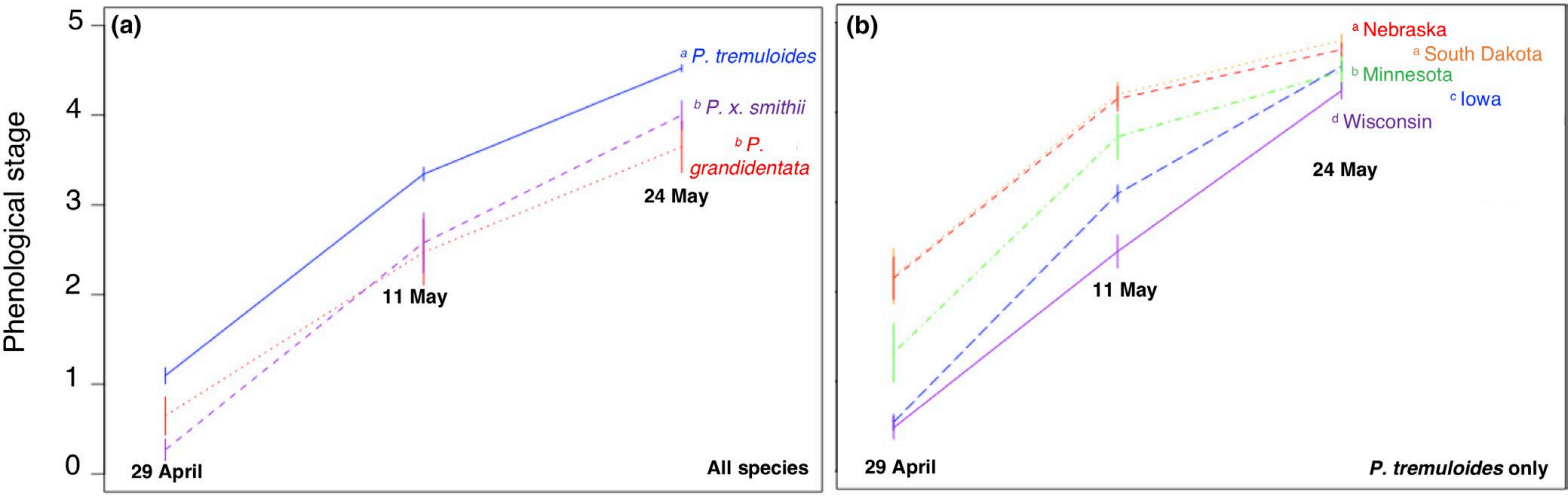
Spring bud break phenology of aspens planted in a common garden in east‐central Minnesota, USA. (a) *Populus tremuloides* (blue) broke bud (reached stage 3; initial leaf emergence) roughly a week before *Populus grandidentata* (red) and their hybrid, *Populus x smithii* (purple). (b) Individuals of *P. tremuloides* varied significantly in their common garden phenology by state of origin. Trees from hot and dry western states (red, orange) showed advanced phenology compared to trees from cool and wet eastern states (green, blue, purple). Superscripts indicate significant (a) species or (b) state differences at the 0.05 level via Tukey test of an ANOVA model including date and either species or state

The leafout phenology of aspens in the common garden varied significantly among taxa and, within *P. tremuloides,* by state of origin (Figure 2; Table 1). *Populus tremuloides* showed advanced phenology relative to *P. grandidentata*, with their hybrid *P. x smithii* more similar to *P. grandidentata* (Figure 2a; *df* = 8, 324; *F* = 18.2, *p* < 0.001). Among *P. tremuloides* individuals, phenology was better explained by state of origin than by any germane climate variables (AHM or NFFD), with eastern populations generally showing later phenology in the common garden (Figure 2b; *df* = 14, 836; *F* = 135.8, *p* < 0.001). For instance, *P. tremuloides* individuals from Wisconsin and Iowa collections were more similar to phenologically slower *P. grandidentata* or hybrid aspens compared to individuals from Nebraska and South Dakota, which leafed out much earlier.

**Table 1:** Mean values of phenological and physiological measurements on a species basis. Values with shared symbols are not distinguishable by Tukey post hoc tests at the 0.05 level

	*Populus grandidentata*	*Populus x smithii*	*Populus tremuloides*
Phenology			
Date of bud break in common garden	19 May^b^	17 May^b^	11 May^a^
Predicted days until bud break after 120 days of winter	28.8^a^	–	24.2^b^
Freezing			
Stem freezing tolerance* @ −15℃ on 21 March (%)	82.7%^a^	77.2%^ab^	71.7%^ab^
Stem freezing tolerance* @ −15℃ on 17 May (%)	56.0%^ab^	55.7%^ab^	47.7%^b^
Stem freezing tolerance* @ −15℃ on 10 October (%)	70.4%^a^	65.5%^ab^	63.8%^b^
Leaf freezing tolerance @ −7℃ (%)	98.8%	97.2%	99.5%
Drought			
Water potential at 50% loss of hydraulic conductivity (MPa)	−1.52^a^	−0.97^ab^	−0.51^b^
Max loss of stem hydraulic conductivity post centrifugation (%)	69.0%^a^	89.7%^b^	93.7%^b^
Leaf osmotic potential (Mpa)	−2.10^a^	−2.06^a^	−2.33^b^
Stomatal pore index (SPI)	0.054^a^	0.085^b^	0.082^b^
Stomatal conductance (g; moles m^−2^ s^−1^)	0.251	0.260	0.199
Photosynthetic rate (*A*_max_; µmoles m^−2^ s^−1^)	13.1	14.4	11.5
Water use efficiency (WUE; *A*_max_/*g*)	54.3	55.4	59.5

* Stem freezing tolerance calculated as 100 less the index of injury calculated to compare the electrolyte leakage from cell rupture at freezing temperatures compared to boiled electrolyte leakage (after Flint et al., 1967). Larger values indicate higher tolerance.

Values with shared superscripts (^a, b, c^) are not distinguishable by Tukey post hoc tests at the 0.05 level.

**Figure 3:**
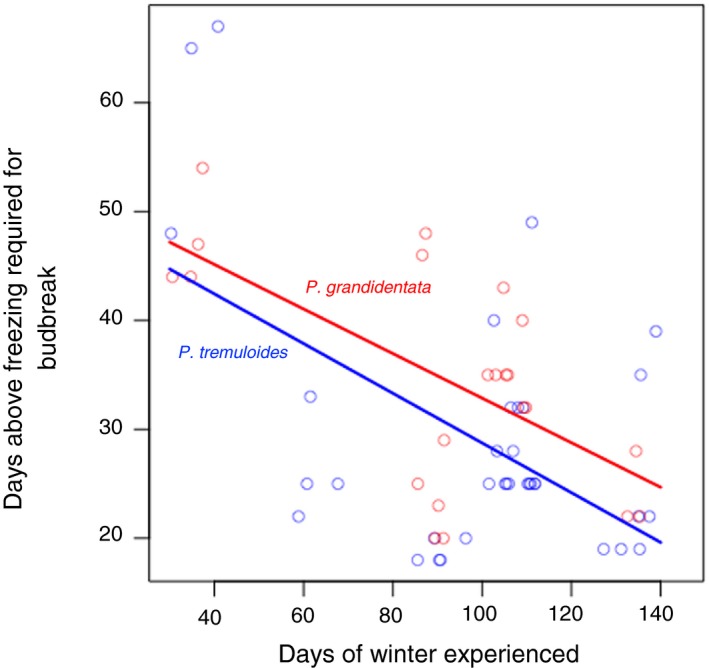
Branches of mature *Populus grandidentata* and *Populus tremuloides* collected from the area of the common garden in east‐central Minnesota on different dates and incubated in warm conditions in the greenhouse differed significantly in the days of warmth they required to break bud. Across a range of simulated winter lengths, *P. tremuloides* stems broke bud about four days earlier

Greenhouse studies of bud break among aspen branches collected on mature trees in central Minnesota showed that *P. tremuloides* required briefer exposure to freezing temperatures for bud break to occur relative to *P. grandidentata,* breaking bud on average 4 days earlier in a controlled environment (Figure 3; Table 1; *df* = 3, 100; *F* = 24.9, *p* < 0.001). Both species reacted similarly to prolonged exposure to winter freezing (no significant interaction), although longer winter duration had a stronger effect on *P. tremuloides* than on *P. grandidentata*.

### 3.2 Stem freezing

**Figure 4:**
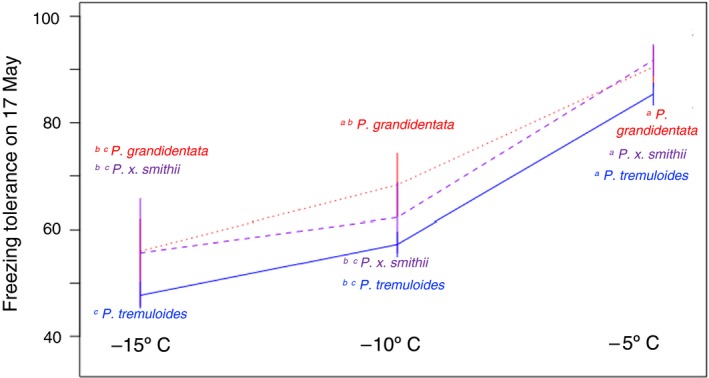
Aspen stem tolerance of experimentally induced freezing depended on freezing temperature and species. *Populus grandidentata* (red) was less vulnerable to freezing at low temperatures (−15° to −10°C) than was *Populus tremuloides* (blue), with their hybrid intermediate (purple). Species did not differ and were relatively invulnerable to freezing at −5°C. Superscripts indicate significant species differences at the 0.05 level via Tukey test of an ANOVA model including freezing temperature and species

Stems of all aspens were relatively tolerant of freezing (at −5 to −15°C) following bud break. *P. grandidentata* and the hybrid *P. x smithii* aspens were more resistant than *P. tremulloides*, experiencing only about 29% damage when frozen in May, compared to 37% damage for *P. tremuloides* averaged across all freezing temperatures according to the Index of Injury calculation (Figure 4; *df* = 5, 183; *F* = 34.8, *p* < 0.001). Colder freezing temperatures, approaching typical minimum temperatures experienced at the common garden, were more damaging to stems than milder freezing temperatures. When frozen in May, stems only experienced 13% damage at −5°C, while sustaining an average of 50% damage at −15°C (Figure 4).

**Figure 5:**
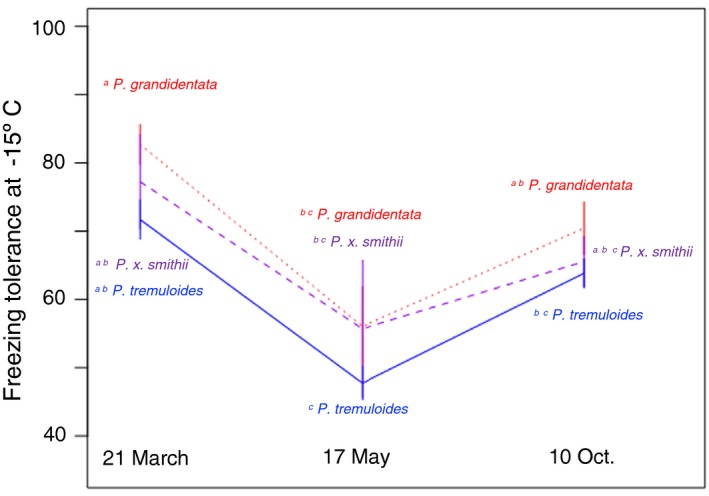
Aspen stem tolerance of experimentally induced freezing at −15°C depended on date of stem collection and species. *Populus grandidentata* (red) was less vulnerable to freezing early and late in the growing season than was *P. tremuloides* (blue), with their hybrid intermediate (purple). Species were most vulnerable to freezing in May, after they had leafed out but prior to acclimation to cool autumn temperatures. Superscripts indicate significant species differences at the 0.05 level via Tukey test of an ANOVA model including stem collection date and species

Timing of freezing events significantly influenced stem damage. Early spring freezes were least damaging to aspen stems, causing an average of only 26% damage, while late‐fall freezes caused slightly more (35%) damage (Figure 5; Table 1). The greatest freezing damage (50%) was exhibited in May, once the growing season had already begun. Freezing during this period tended to cause more damage across all taxa and temperatures (*df* = 2, 198; *F* = 32.0, *p* < 0.001). There was no strong influence of state or climate of origin on freezing damage to stems of *P. tremuloides*; freezing damage was not more or less severe in individuals from warmer climates.

### 3.3 Leaf freezing

Aspen leaves were generally resistant to freezing, rarely losing more than 10% of their maximum quantum yield following a freeze event and recovering after freezing to Fv/Fm values of 0.8, indicating near‐full function (Appendix S4). Taxa did not differ significantly in loss of photosynthetic function following freezing, and *P. tremuloides* individuals did not vary by state or climate of origin.

### 3.4 Stem hydraulic conductance and vulnerability to drought embolism

**Figure 6:**
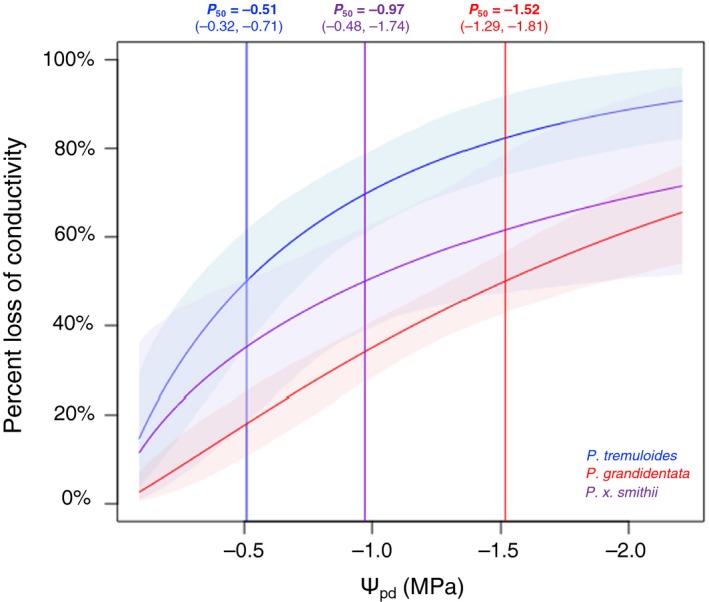
*Populus grandidentata* (red) showed lower loss of native stem hydraulic conductivity with increasing xylem tension (negative water potential) than did *Populus tremuloides* (blue). Hybrid *Populus x smithii* (purple) was intermediate. Shading around curves indicates 95% confidence intervals. Vertical lines show mean P_50_ (water potential at which 50% loss of conductivity occurred) for each species with values and confidence intervals provided above the plot

*Populus tremuloides* was more vulnerable to cavitation with spinning than was *P. grandidentata*, with the *P. x smithii* hybrid intermediate between them (Figure 6; Table 1). The water potential at which 50% loss of stem hydraulic conductivity occurred (P_50_) was significantly less negative in *P. tremuloides* (−0.51 MPa) than in *P. x smithii* (−0.97 MPa) or *P. grandidentata* (−1.52 MPa). Native embolism did not differ among species, but at −2.2 MPa, the maximum tension imposed, average PLC was significantly lower (68%) for *P. grandidentata* than for *P. tremuloides* (93%), with *P. x smithii* (74%) intermediate between its parental species (*df* = 2, 23; *F* = 3.64, *p* = 0.042).

### 3.5 Leaf osmotic potential and stomatal pore index

**Figure 7:**
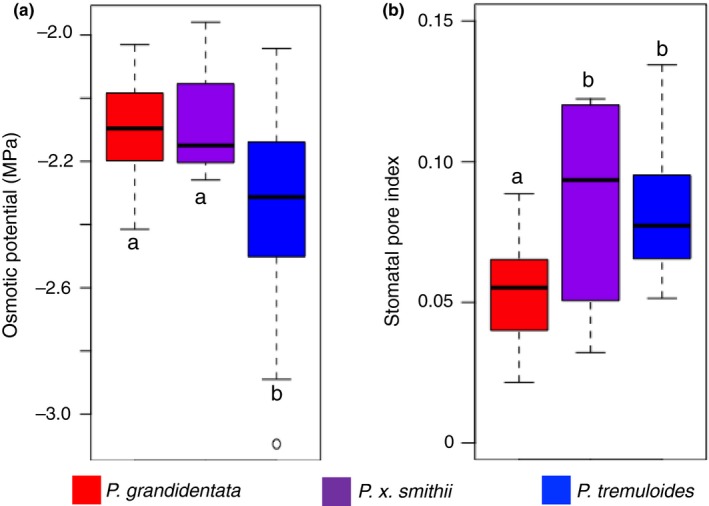
Species differed significantly in leaf traits related to water use and drought vulnerability. (a) *Populus tremuloides *(blue) leaves had a significantly more negative osmotic potential, allowing increased water access during drought (a drought avoidance strategy) than *Populus grandidentata *(red). (b) Compared to *P. tremuloides, P. grandidentata had lower* stomatal pore index (SPI), which allows leaves to minimize water loss. Hybrid *Populus x smithii* (purple) leaves showed lower negative osmotic potential similar to *P. grandidentata *but higher SPI similar to *P. tremuloides*. Letters indicate significant differences at the 0.05 level from a Tukey post hoc test

The parental aspen species differ in two heritable traits controlling water use and drought tolerance. *Populus tremuloides* had a more negative osmotic potential than *P. grandidentata* (Figure 7a; *df* = 2, 76; *F* = 4.56, *p* = 0.013) but a higher stomatal pore index (Figure 7b; *df* = 2, 77; *F* = 5.72, *p* = 0.005), indicating that it had a greater a greater capacity to absorb water in dry conditions, but a larger stomatal pore surface area, associated with higher leaf hydraulic conductance (Sack et al., 2003), and lower ability to prevent water loss and desiccation (Cavender‐Bares, Sack, & Savage, 2007). The hybrid *P. x smithii* showed less negative osmotic potential, like *P. grandidentata*, but high SPI, like *P. tremuloides* (Figure 7a; Table 1), indicating lower water absorption capability and low resistance to water loss.

### 3.6 Gas exchange and water use efficiency

**Figure 8:**
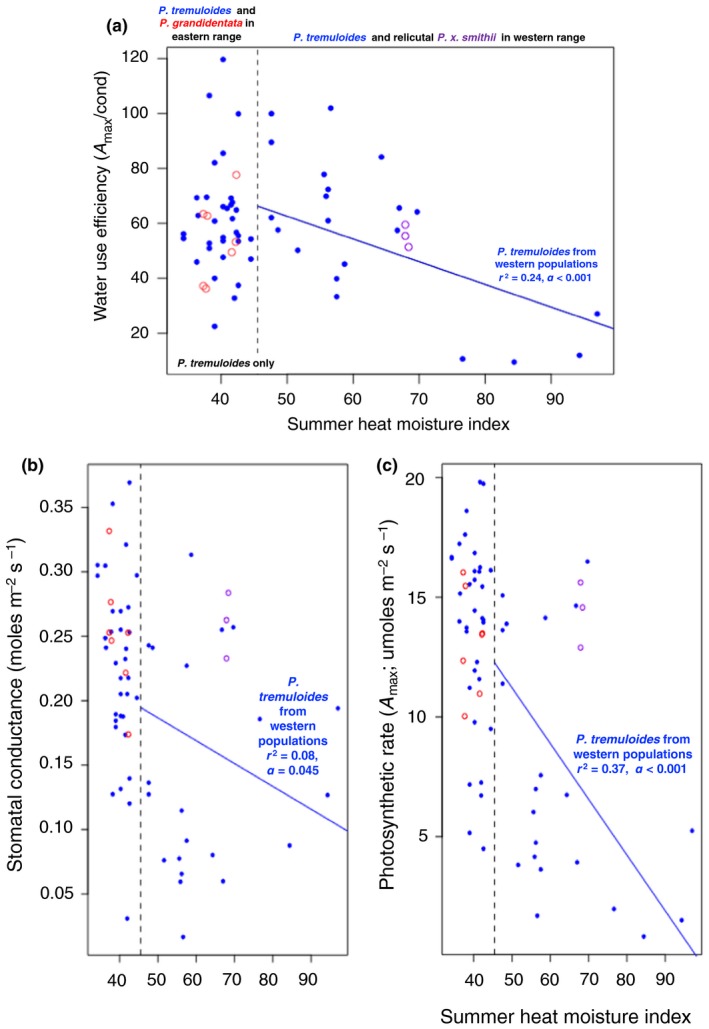
(a) Intrinsic water use efficiency (WUEi; light saturated *A*_max_/*g*_s_) in the common garden did not vary by species or in plants collected in more mesic, eastern sites. However, when only *Populus tremuloides* individuals from drier, hotter western sites (those with a high summer heat moisture index) were considered, plants from the driest and hottest sites had lower WUE in the common garden. The dashed line indicates the highest summer heat moisture index value recorded for a *Populus grandidentata* observation in the GBIF database. *Populus tremuloides* individuals collected from sites with a higher Index value than this are included in the regression. (b) Observed light saturated stomatal conductance (*g*_s_) and (c) photosynthetic rate (*A*_max_), in the common garden did not vary by species (red: *P. grandidentata*, purple: *Populus x smithii*, blue: *P. tremuloides*). There was no relationship between *g*_s_ or *A*_max_ and climate (summer heat moisture index) in the range of *P. grandidentata* and the eastern part of the range of *P. tremuloides*. Yet in the western part of the range of *P. tremuloides*, *A*_max_ declined precipitously (and *g*_s_ did to a lesser extent) contributing to a significantly lower WUEi of plants from warmer and drier climates

Intrinsic water use efficiency (WUEi) in the common garden did not vary by taxon or by state, but roughly 10% of the variation in gas exchange (light saturated A and *g*_s_) was associated with climate of origin (AHM, increasing as conditions become more hot and dry) (Figure 8a). Plants from hotter, drier climates showed lower WUEi (as well as lower A (Figure 8b) and *g*_s_(Figure 8c)) in the common garden (*df* = 1, 62; *F* = 8.57, *p* = 0.005). This relationship was stronger when only *P. tremuloides* from the western, hot/dry region of the study population was considered (explains 24% of variation in WUEi); there was no relationship between climate of origin and WUEi in the eastern/mesic individuals of *P. tremuloides* (Figure 8a).The greater WUE of plants originating from warmer and drier climates is not readily explained by patterns in either *g*_s_ or *A*_max_ (correlations between WUE and each of the latter variables are insignificant; Figure 8b and c; Table 1).

## 4 DISCUSSION

*Populus tremuloides* and *P. grandidentata* show different strategies for surviving drought and freezing, with *P. x smithii* sharing qualities of both parents. Based on current species ranges, we expected *P. tremuloides* to vary in its response to these stressors more than the other two taxa. Delayed bud break and leafout phenology play an important role in the avoidance of potential late‐spring freeze events in *P. grandidentata,* and its stems were not as injured in our freezing experiments compared to *P. tremuloides*. These factors correspond to a freezing avoidance strategy in *P. grandidentata* and a freezing tolerance strategy in *P. tremuloides*. In contrast, given its lower stem vulnerability to embolism, *P. grandidentata* exhibits greater drought tolerance than *P. tremuloides*. Meanwhile, the more negative osmotic potential of *P. tremuloides* allows it to access water more readily during drought, indicating a drought avoidance strategy. The two parental species thus contrast in important ways in their survival mechanisms in response to climatic stress, with *P. tremuloides* tending toward freezing tolerance but drought avoidance and *P. grandidentata* tending toward freezing avoidance and drought tolerance.

It is clear from other comparisons of traits and genomic architecture among parental species and hybrids across the genus *Populus*, that nuclear and chloroplast variation play a large role in determining hybrid performance (Christe et al., 2016; Dillen, Marron, Koch, & Ceulemans, 2008; Gompert, Mandeville, & Buerkle, 2017; Jiang et al., 2016; Lexer et al., 2005; Taylor, Larson, & Harrison, 2015) As sister species and the only native North American aspens in the section *Populus* (*Leuce*), *P. grandidentata* and *P. tremuloides* may have differentiated along drought/cold niche axes. Following the last ice age, *P. tremuloides* may have been preadapted to dry/cold conditions, so its range has expanded as it has followed glaciers. (Jaramillo‐Correa, Beaulieu, Khasa, & Bousquet, 2009). Our results are consistent with observations about low hydraulic conductance rates, bud break timing, and stem embolism in temperate deciduous trees of similar wood anatomy where species that flushed leaves earlier had higher rates of embolism (Wang, Ives, & Lechowicz, 1992). The connection between early spring leaf flushing and traits associated with stress response is common to both drought and freezing (Bohnert, Nelson, & Jensen, 1995) so these sister species may be preadapted to tolerate both of these stresses.

*Populus x smithii* shares freezing avoidance traits with *P. grandidentata*, but freezing is not currently, nor is it projected to be, a big challenge for our focal population. The freezing temperatures imposed in our experiments that resulted in a high index of injury are much lower than climate records for occasional spring freeze events for the Niobrara River Valley of Nebraska. In fact, the freezing avoidance traits of *P. x smithii* may put it at a disadvantage in a warming climate where protection from freezing damage comes with tradeoffs for growth and productivity (Koehler et al., 2012; Savage & Cavender‐Bares, 2013).

In contrast to the similarity between the hybrid and one parent for freezing avoidance, *P. x smithii* shares drought tolerance and avoidance traits with both parents. The ability to regulate leaf osmotic potential allows plants to deal with both the stress of drought and freezing (Sakai & Larcher, 1987) and the osmotic potential of *P. x smithii* is more similar to *P. grandidentata* and less negative than *P. tremuloides*. Solutes in the cell can protect membranes and proteins while also helping to maintain turgor and there can be strong selection for low (more negative) osmotic potential in summer drought or winter cold (Callister et al., 2008). Unlike regionally sympatric *P. tremuloides*, *P. x smithii* has less negative osmotic potential and does not regulate stomatal conductance like the western *P. tremuloides*, where much lower water use efficiency (WUE) was observed. Typically high WUE and low stomatal conductance are characteristics of drought tolerant plants, whereas low WUE and high stomatal conductance are seen in drought avoiding species that maximize growth before drought intensifies (Heschel & Riginos, 2005). *P. x smithii* has a lower stomatal pore index (SPI) than *P. tremuloides*, similar to *P. grandidentata*. SPI combines density and size of stomata and is usually correlated with stomatal conductance (Sack et al., 2003). Species or populations with a lower SPI tend to have lower stomatal conductance and higher WUE.

The greater loss of stem hydraulic conductivity with increasing xylem tension (more negative water potential) in *P. tremuloides* provided evidence that is the most vulnerable of the three taxa to cavitation/embolism. Its lack of drought tolerance in the stem and the higher stomatal pore area in the leaves are both indicative of drought avoidance. This characterization is further supported by its more negative osmotic potential that promotes greater water access during drought. In contrast, *P. grandidentata*'s more embolism‐resistant stems and leaves with low stomatal pore area that minimize water loss are indicative of a drought tolerance strategy. The hybrid is intermediate with the less negative osmotic potential of the drought tolerant parent but higher stomatal pore index of the drought avoidant parent. It thus has limited ability to minimize water loss without the advantage of higher leaf osmotic concentration that enables the plant to access water at more negative water potentials. The intermediate resistance to stem embolism/cavitation in *P. x smithii* could counteract the water losing responses in the leaves. However, in a warmer and drier climate, the trait combination of the hybrid is likely to result in rapid loss of leaf turgor pressure, leaf wilting, and hence loss of photosynthetic function and ultimately productivity (Zhu et al., 2018).

The persistence of this unique population of *P. x smithii* along the NNSR is already in question because of observations of decline and symptoms of Sudden Aspen Dieback (SAD) (Robertson, 2015; Robertson et al., 2018). IPCC Projections of climate change predict 2–6°C temperature increase from 2025–2034 compared to 1961–1990 and increased precipitation (0%–20%) that will be offset by increasing temperatures, resulting in further drought stress. Warmer and shorter winters will likely not have any negative effects on *P. x smithii* because spring freeze events are unlikely to be cold enough, but successful management of the NRV aspens may require greater intervention than has been attempted to date.

## Data Availability

All data are accessible in the Cedar Creek Ecosystem Science Reserve data repository: https://www.cedarcreek.umn.edu/research/data/experiment?e298.
