# Supplementary material for: Drought and freezing vulnerability of the isolated hybrid aspen Populus x smithii relative to its parental species, P. tremuloides and P. grandidentata
Source: Ecol Evol. 2019 Jun 25;9(14):8062–74. doi: 10.1002/ece3.5364 (PMC6662423; doi:10.1002/ece3.5364)
Supplement: Supplementary file 3 [file ECE3-9-8062-s003.docx]

Appendix S3: Estimation of climate envelopes

To construct climate envelopes, we extracted all occurrences of *P. tremuloides* (N = 3070) and *P. grandidentata* (N = 628) from the Global Biodiversity Information Facility (GBIF.org 2017). We then queried the WorldClim database (Vers. 1.4; Hijmans et al. 2005) to retrieve climate data for each latitude/longitude pair associated with an aspen occurrence. We were able to assemble climate data for a subset of occurrences of both *P. tremuloides* (N = 1732) and *P. grandidentata* (N = 274) and used these data to approximate climate envelopes for each species. Ultimately, we compared the climatic envelopes of each species with reference to bioclimatic variables BIO1 (mean annual temperature; °C) and BIO12 (mean annual precipitation; mm). We visualized these envelopes using the *stat_density2d* tool in the “ggplot” function of the ggplot2 R package (alpha = 0.5).

GBIF.org (1 March 2017) GBIF Occurrence Download [P. grandidentata: <https://doi.org/10.15468/dl.eai95e> and P. tremuloides: <https://doi.org/10.15468/dl.idmfts>]
