# Supplementary material for: Drought and freezing vulnerability of the isolated hybrid aspen Populus x smithii relative to its parental species, P. tremuloides and P. grandidentata
Source: Ecol Evol. 2019 Jun 25;9(14):8062–74. doi: 10.1002/ece3.5364 (PMC6662423; doi:10.1002/ece3.5364)

Appendix S4: Aspen leaves lost very little maximum quantum yield (defined as values of Fv/Fm lower than 0.8) following freezing at -7°C. Species did not vary in their vulnerability.


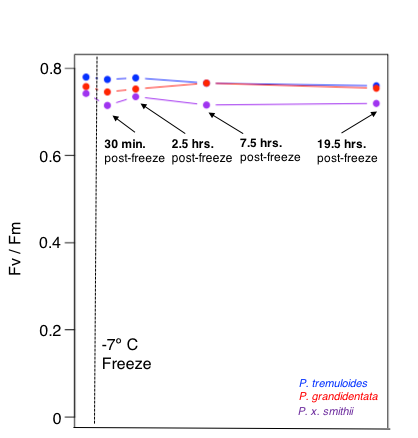

Supplement: Supplementary file 4 [file ECE3-9-8062-s004.docx]
